# Antimicrobial susceptibilities of aerobic and facultative gram-negative bacilli isolated from Chinese patients with urinary tract infections between 2010 and 2014

**DOI:** 10.1186/s12879-017-2296-x

**Published:** 2017-03-06

**Authors:** Qiwen Yang, Hui Zhang, Yao Wang, Zhipeng Xu, Ge Zhang, Xinxin Chen, Yingchun Xu, Bin Cao, Haishen Kong, Yuxing Ni, Yunsong Yu, Ziyong Sun, Bijie Hu, Wenxiang Huang, Yong Wang, Anhua Wu, Xianju Feng, Kang Liao, Yanping Luo, Zhidong Hu, Yunzhuo Chu, Juan Lu, Jianrong Su, Bingdong Gui, Qiong Duan, Shufang Zhang, Haifeng Shao, Robert E. Badal

**Affiliations:** 1 Department of Clinical Laboratory, Peking Union Medical College Hospital, Peking Union Medical College, Chinese Academy of Medical Sciences, Beijing, 100730 China; 2 Department of Respiratory and Critical Care Medicine, Clinical Microbiology and Infectious Disease Lab., China-Japan Friendship Hospital, Beijing, 100029 China; 3 Department of Microbiology, The First Affiliated Hospital of Zhejiang University, Hangzhou, 310003 China; 4 Division of Microbiology, Ruijin Hospital, School of Medicine, Shanghai Jiaotong University, Shanghai, 200025 China; 5 Department of Infectious Diseases, SirRunRun Shaw Hospital, School of Medicine, Zhejiang University, Hangzhou, 310016 China; 6 Department of Laboratory Medicine, Tongji Hospital, Tongji Medical College, Huazhong University of Science and Technology, Wuhan, 430030 China; 7 Division of Microbiology, Zhongshan Hospital of Fudan University, Shanghai, 200032 China; 8 Division of Microbiology, The First Affiliated Hospital of Chongqing Medical University, Chongqing, 400016 China; 9 Department of Laboratory Medicine, Shandong Provincial Hospital Affiliated to Shandong University, Jinan, 250021 China; 10 Infection control center, Xiangya Hospital, Central South University, Changsha, 410008 China; 11 Division of Microbiology, The First Affiliated Hospital of Zhengzhou University, Zhenzhou, 450052 China; 12 Division of Microbiology, The First Affiliated Hospital, Sun Yat-Sen University, Guangzhou, 510080 China; 13 Department of Microbiology, The Chinese PLA General Hospital, Beijing, 100853 China; 14 Division of Microbiology, Tianjin Medical University General Hospital, Tianjing, 300052 China; 15 Division of Microbiology, The First Affiliated Hospital of Chinese Medical University, Shenyang, 110001 China; 16 Department of Clinical Laboratory, The First Affiliated Hospital of Harbin Medical University, Harbin, 150001 China; 17 Department of Clinical Laboratory, Beijing Friendship Hospital of Capital Medical University, Beijing, 100020 China; 18 Clinical Laboratory, The Second Affiliated Hospital of Nanchang University, Nanchang, 330006 China; 19 Microbiology Lab, Jilin Province People’s Hospital, Changchun, 130021 China; 20 Division of Microbiology, Haikou People’s Hospital, Haikou, 570208 China; 21 Division of Microbiology, General Hospital of Nanjing Military Command, Nanjing, 210002 China; 22 Division of Microbiology, International Health Management Associates, Schaumburg, IL 60173-3817 USA

**Keywords:** Urinary tract infections, Extended spectrum beta-lactamases (ESBLs), Carbapenems, Antimicrobial resistance

## Abstract

**Background:**

The objective of this study was to investigate the distribution and susceptibility of aerobic and facultative Gram-negative bacilli isolated from Chinese patients with UTIs collected within 48 h (community acquired, CA) or after 48 h (hospital acquired, HA) of hospital admission.

**Methods:**

From 2010 to 2014, the minimum inhibitory concentrations (MICs) of 12 antibiotics for 4,332 aerobic and facultative Gram-negative bacilli, sampled in 21 hospitals in 16 cities, were determined by the broth microdilution method.

**Results:**

*Enterobacteriaceae* composed 88.5% of the total isolates, with *Escherichia coli* (*E. coli*) (63.2%) the most commonly isolated species, followed by *Klebsiella pneumoniae* (*K. pneumoniae*) (12.2%). Non-*Enterobacteriaceae* accounted for only 11.5% of all isolates and included mainly *Pseudomonas aeruginosa* (*P. aeruginosa*) (6.9%) and *Acinetobacter baumannii* (*A. baumannii*) (3.3%). Among the antimicrobial agents tested, the susceptibility rates of *E.coli* to the two carbapenems, ertapenem and imipenem as well as amikacin and piperacillin-tazobactam ranged from 92.5 to 98.7%. Against *K. pneumonia,* the most potent antibiotics were imipenem (92.6% susceptibility), amikacin (89.2% susceptibility) and ertapenem (87.9% susceptibility).

Although non-*Enterobacteriaceae* did not show high susceptibilities to the 12 common antibiotics, amikacin exhibited the highest in vitro activity against *P. aeruginosa* over the 5-year study period, followed by piperacillin-tazobactam, imipenem, ceftazidime, cefepime, ciprofloxacin, and levofloxacin. The Extended Spectrum Beta-Lactamase (ESBL) rates decreased slowly during the 5 years in *E. coli* from 68.6% in 2010 to 59.1% in 2014, in *K. pneumoniae* from 59.7 to 49.2%, and in *Proteus mirabilis* (*P. mirabilis*) from 40.0 to 26.1%. However, the ESBL rates were different in 5 regions of China (Northeast, North, East, South and Middle-China).

**Conclusion:**

*E. coli* and *K. pneumonia* were the major pathogens causing UTIs and carbapenems and amikacin retained the highest susceptibility rates over the 5-year study period, indicating that they are good drug choices for empirical therapies, particularly of CA UTIs in China.

## Background

Several national and international surveillance programs have been initiated for monitoring susceptibilities of clinically important pathogens in urinary tract infections (UTIs) [1–3]. The Study for Monitoring Antimicrobial Resistance Trends (SMART) is a surveillance program designed to monitor globally susceptibilities of aerobic and facultative Gram-negative bacilli collected from intra-abdominal infections and UTIs (initiated in 2002) [4]. UTIs are frequently encountered in clinical practice and include uncomplicated and complicated pyelonephritis, ureteritis, cystitis and urethritis [5]. The etiologies of these infections arise from Gram-negative bacilli, especially *Enterobacteriaceae,* and some Gram-positive bacteria [6]. During the last decade, multidrug-resistant Gram-negative Enterobacteriaceae have become a challenge for physicians [7] and particularly *E. coli* and *K. pneumonia* strains isolated from UTIs have been reported to increasingly produce ESBLs in the recent years [8–10]. The choice of an empiric UTI antimicrobial therapy should be based on knowledge of the pathogen distribution and the resistance extent of common microorganisms, in addition to hospital-specific resistance patterns particularly for HA infections. This study, as part of the global SMART project, focused on ESBL-producing rates of UTI isolates from 21 centers in 16 Chinese cities between 2010 and 2014 and on UTI derived sample resistance rates against carbapenems, a combination of drugs containing penicillins with β-lactamase inhibitors, a cephamycin, an aminoglycoside, 3rd and 4^th^generation cephalosporins as well as 2nd generation fluoroquinolones, in order to provide guidance for antimicrobial therapies of IAIs.

## Methods

### Clinical isolates

During our study period (2010–2014), a total of 4,332 aerobic and facultative Gram-negative bacilli were consecutively isolated from patients with UTIs in 21 hospitals sited in 16 Chinese cities (Beijing, Shanghai, Hangzhou, Nanjing, Shenyang, Tianjin, Wuhan, Changsha, Jinan, Zhengzhou, Guangzhou, Nanchang, Haikou, Harbin, Changchun and Chongqing).

All isolates were cultured from specimens collected from patients who met both clinical and laboratory criteria of urinary tract infections (3,994 from clean catch midstream urine, 154 from urinary bladder, 136 from ureter, 29 from kidney, 13 from urethra, 6 from prostate). Duplicate isolates (same species and genus from one patient) were excluded.

Standard methods were used by the participating clinical microbiology laboratories for initial bacteria identification, and re-identification was carried out by a central laboratory (Peking Union Medical College Hospital) using Vitek 2 Compact (2010–2011) (Biomerieux, France) and MALDI-TOF MS (2012–2014) (Vitek MS, Biomerieux, France).

Isolates were considered to be community-associated (CA) if they were recovered from a specimen taken less than 48 h after the patient was admitted to a hospital, and hospital-associated (HA) if the specimen was taken 48 or more hours after hospital admission, as previously described [11].

### Antimicrobial susceptibility test method

Minimum inhibitory concentration (MIC) determinations were performed in a central lab using dehydrated MicroScan broth microdilution panels (Siemens Medical Solutions Diagnostics (West Sacramento, CA) according to Clinical and Laboratory Standards Institute (CLSI) guidelines [12] and susceptibility interpretations were based on clinical CLSI breakpoints [13]. Twelve commonly used antimicrobial agents for UTI treatments were analyzed namely, imipenem (IPM), ertapenem (EPM), ceftriaxone (CRO), cefotaxime (CTX), ceftazidime (CAZ), cefoxitin (FOX), cefepime (FEP), piperacillin-tazobactam (TZP), ampicillin-sulbactam (SAM), amikacin (AMK), ciprofloxacin (CIP) and levofloxacin (LVX). For each batch of MIC testing, the reference strains *E. coli* ATCC 25922, *P. aeruginosa* ATCC 27853 and *K. pneumonia* ATCC 700603 were used as quality controls. Results were only included in the analysis when corresponding quality control isolate test results were in accordance with CLSI guidelines and therefore within an acceptable range.

### Extended-spectrum β-lactamases (ESBLs) detection

Phenotypic identification of ESBL production in *E.coli, K. pneumonia, Klebsiella oxytoca* (*K. oxytoca*)*,* and *P. mirabilis* was carried out according to CLSI recommended methods [13]. If cefotaxime or ceftazidime MICs were ≥ 2 μg/mL, the MICs of cefotaxime + clavulanic acid (4 μg/mL) or ceftazidime + clavulanic acid (4 μg/mL) were comparatively determined. ESBL production was defined as a ≥ 8-fold decrease in MICs for cefotaxime or ceftazidime tested in combination with clavulanic acid, compared to their MICs without clavulanic acid.

### Statistical analysis

The susceptibility of all gram-negative isolates combined was calculated using breakpoints appropriate for each species and assuming 0% susceptible for species with no breakpoints for any given drug. Ninety-five percent confidence intervals were calculated using the adjusted Wald method; linear trends of ESBL rates in different years were assessed for statistical significance using the Cochran-Armitage test and comparison of ESBL rates in 6 different geographic areas were assessed using Chi-square test. *P* values < 0.05 were considered statistically significant.

## Results

### Distribution of organisms from urinary tract infection

A total of 4,332 isolates were collected from UTIs between 2010 and 2014. The highest distribution of bacteria was *E. coli,* which accounted for 63.2% (2,737 strains), followed by *K. pneumonia* (12.2%, 529 strains) and *P. aeruginosa* (6.9%, 297 strains) (Table 1)*.* We also investigated the distribution of strains from HA (*n* = 2765, 72.16%) and CA (*n* = 1039, 27.11%) (*P* **<** 0.0001) infections, but most of the isolates were sampled from HA infections (62.59–80.42%) (Table 1). *Enterobacteriaceae* were present in the majority of isolates and accounted for 88.5%, including mainly *E.coli* (63.2%), followed by *K. pneumonia* (12.2%), *P. mirabilis* (3.4%) and *Enterobacter cloacae* (*E. cloacae*) (3.3%), while others were present at a rate < 1.3%. Non-*Enterobacteriaceae* accounted for only 11.5% of all isolates and included mainly *P. aeruginosa* (6.9%) and *Acinetobacter baumannii* (*A. baumannii*) (3.3%).

**Table 1:** Distribution of the UTI pathogens in China between 2010 and 2014

	Total	CA (n/% of total)	HA (n/% of total)	Not identified (n/% of total)	*P*-value
*Enterobacteriaceae*	3,832	1,039 (27.11)	2,765 (72.16)	28 (0.73)	<0.0001
*Escherichia coli*	2,737	739 (27.00)	1,976 (72.20)	22 (0.80)	<0.0001
*Klebsiella pneumoniae*	529	129 (24.39)	398 (75.24)	2 (0.38)	<0.0001
*Proteus mirabilis*	147	54 (36.73)	92 (62.59)	1 (0.68)	0.011
*Enterobacter cloacae*	141	39 (27.66)	101 (71.63)	1 (0.71)	<0.0001
*Citrobacter freundii*	54	11 (20.37)	43 (79.63)	0 (0.00)	0.0003
*Klebsiella oxytoca*	51	18 (35.29)	33 (64.71)	0 (0.00)	0.1205
*other*	173	49 (28.32)	122 (70.52)	2 (1.16)	<0.0001
*Non-Enterobacteriaceae*	500	105 (21.00)	391 (78.2)	4 (0.8)	<0.0001
*Pseudomonas aeruginosa*	297	65 (21.89)	231 (77.78)	1 (0.34)	<0.0001
*Acinetobacter baumannii*	143	26 (18.18)	115 (80.42)	2 (1.40)	<0.0001
*other*	60	14 (23.33)	45 (75.00)	1 (1.67)	<0.0001
All	4,332	1,144 (26.41)	3,156 (72.85)	32 (0.74)	<0.0001

Not identified: A total of 32 isolates lacked partial demographic information and could not be identified as CA or HA isolates. They were not included in further analyses

#### In vitro susceptibility of Enterobacteriaceae and non-Enterobacteriaceae during 2010–2014

**Fig. 1:**
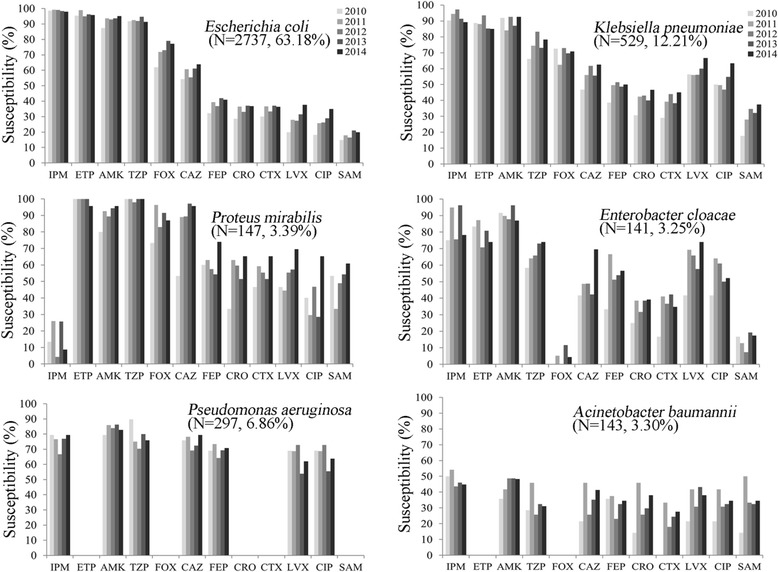
Trends over time in the susceptibility of isolates from UTIs to antimicrobial agents in China. *EPM, ertapenem; IPM, imipenem; AMK, amikacin; TZP, piperacillin-tazobactam; FOX, cefoxitin; FEP, cefepime; CAZ, ceftazidime; CRO, ceftriaxone; CTX, cefotaxime; LVX, levofloxacin; CIP, ciprofloxacin; SAM, ampicillin-sulbactam. Note: The data of ETP FOX CRO and CTX susceptibilities for *P. aeruginosa* and ETP as well as FOX sensitivities for *A. baumannii* were not shown because of lack of corresponding breakpoints

Among the 12 analyzed antimicrobial agents, the susceptibility rates of ertapenem and imipenem against *E. coli* over 5 years were 96.4% (2,639/2,737) and 98.7% (2,702/2,737), with MIC_90_ values of 0.25 μg/mL for both drugs. Most *E.coli* isolates remained susceptible to amikacin (92.8%) and piperacillin-tazobactam (92.51%). However, the susceptibilities to third- and fourth-generation cephalosporins were relatively low, with rates of 58.5, 38.2, 34.6 and 34.4% for ceftazidime (CAZ), cefepime (FEP), cefotaxime (CTX) and ceftriaxone (CRO), respectively. The susceptibility rates of *E. coli* to fluoroquinolones and ampicillin-sulbactam were also less than 30 and 20%, respectively (Fig. 1, Table 2).

**Table 2:** Susceptibilities of UTI pathogens isolated between 2010 and 2014

	Antibiotics	S%	MIC50 (μg/ml)	MIC90 (μg/ml)
*Escherichia coli*	IPM	98.72	0.12	0.25
	ETP	96.42	≤0.03	0.25
	AMK	92.88	≤4	16
	TZP	92.51	≤2	16
*Klebsiella pneumoniae*	IPM	92.63	0.25	1
	AMK	89.22	≤4	>32
	ETP	87.9	≤0.03	1
	TZP	75.8	≤2	>64
*Proteus mirabilis*	ETP	99.32	≤0.03	0.06
	TZP	99.32	≤2	4
	AMK	91.16	8	16
	CAZ	88.44	≤0.5	8
*Enterobacter cloacae*	AMK	90.07	≤4	16
	IPM	85.11	0.5	2
	ETP	78.72	0.12	4
	TZP	67.38	4	>64
*Pseudomonas aeruginosa*	AMK	84.18	8	>32
	TZP	76.43	4	>64
	IPM	74.75	1	>8
	CAZ	74.41	4	64
*Acinetobacter baumannii*	IPM	46.85	8	>8
	AMK	46.15	>32	>32
	LVX	36.36	>4	>4
	CAZ	34.27	64	>128

Against *K. pneumonia,* the most potent antibiotics were imipenem (92.6% susceptibility), amikacin (89.2% susceptibility) and ertapenem (87.9% susceptibility), with MIC_90_ values of 1 μg/mL, > 32 μg/mL and 1 μg/mL, respectively. Piperacillin-tazobactam was the fourth most active agent, with a susceptibility of 75.8%. The susceptibility rates of other antibiotics ranged from 30.6% (ampicillin-sulbactam) to 67.5% (cefoxitin) (Fig. 1, Table 2,).

**Fig. 2:**
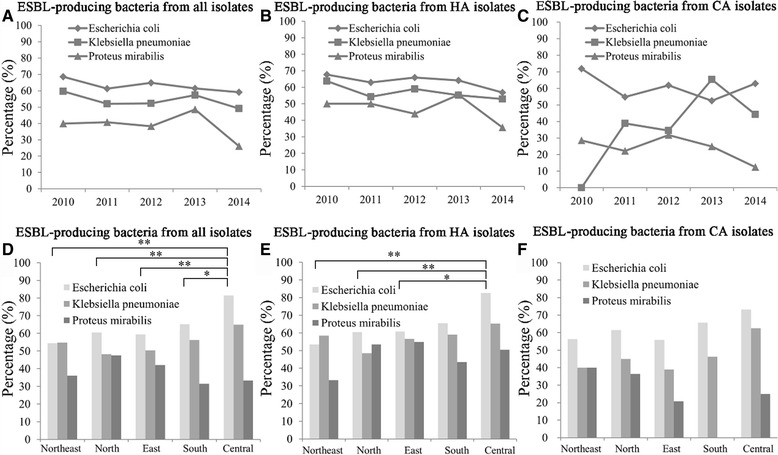
ESBL rate of *Escherichia coli, Klebsiella pneumoniae* and *Proteus mirabilis* from UTIs in different regions and years in China (SMART 2010–2014). **a**-**c** ESBL rates of all samples, **b** ESBL rates of HA UTI isolates, **c** ESBL rates of CA UTIs. **d** ESBL rates of all samples, **e** HA UTI isolates and **f** CA UTI isolates collected between 2010 and 2014 in the indicated Chinese regions. * *P* < 0.05; ** *P* < 0.01

Against *P. mirabilis*, antimicrobial agents with > 90% susceptibility rates included ertapenem (99.3%), piperacillin-tazobactam (99.3%) and amikacin (91.2%), but in HA infections, a > 90% susceptibility rate was found for ceftazidime (90.2%). Cephalosporin susceptibility rates were 55.8–88.4% whereas fluoroquinolones exhibited 41.5–55.1% activity. Imipenem had poor activity against *P. mirabilis* isolates, with a mean susceptibility rate of only 15.0% in both CA and HA derived isolates (Figs. 1 and 2, Table 2).

Antimicrobial resistance in *Enterobacter cloacae* was more pronounced than in *E. coli* and *K. pneumonia.* The antimicrobial agents with susceptibility rates of > 80% were amikacin (90.1%) and imipenem (85.1%) over the 5-year study period. Particularly in 2014, ertapenem and piperacillin-tazobactam susceptibility rates in HA infections dropped to 53.9%, whereas CA UTIs were still 100% susceptible to both antibiotics (Fig. 2). However, ertapenem was the third most active agent with susceptibilities of 78.7% in all isolates, followed by piperacillin-tazobactam (67.4%), levofloxacin (64.5%), ciprofloxacin (56.7%) and cefepime (55.3%) (Fig. 1, Table 2).

Although non-*Enterobacteriaceae* did not show high susceptibilities to the 12 common antibiotics, amikacin exhibited the highest in vitro activity against *P. aeruginosa*, with a susceptibility rate of 84.2% over the 5-year study period, followed by piperacillin-tazobactam, imipenem, ceftazidime, cefepime, ciprofloxacin, and levofloxacin. (Figure 1, Table 2).

*A. baumannii* was the second most frequently isolated non-fermentative Gram-negative bacillus, comprising 3.3% (143/4,332) of all UTIs. The most active agents against *A. baumannii* were imipenem and amikacin, with susceptibility rates of 46.9 and 46.2%, respectively over the entire study period. The other analyzed agents were less effective, with susceptibility rates of < 40% (Fig. 1, Table 2).

#### The trend of extended spectrum beta-lactamases (ESBL) –producing bacteria occurrence in UTIs from 2010 to 2014

Figure 2a-c shows the frequency of ESBL-producing *E.coli, K. pneumonia, K. oxytoca and P. mirabilis* strains over the study period. The percentage of ESBL positive *E. coli* isolates decreased from 68.6% in 2010 to 59.1% in 2014, while the ESBL rate in *K. pneumonia* decreased from 59.7 to 49.2% and in *P. mirabilis* from 40.0 to 26.1% during the 5-year study period. The susceptibility differences to ertapenem and imipenem between ESBL and non-ESBL producing strains were generally small, but were greater for other agents, particularly for the third- and fourth-generation cephalosporins, including ceftriaxone (1.1% against ESBL-producing isolates vs 91.0% against ESBL-non-producing isolates), ceftazidime (38.4% vs 93.5%) and cefepime (4.5% vs 96.7%) (data not shown).

**Fig. 3:**
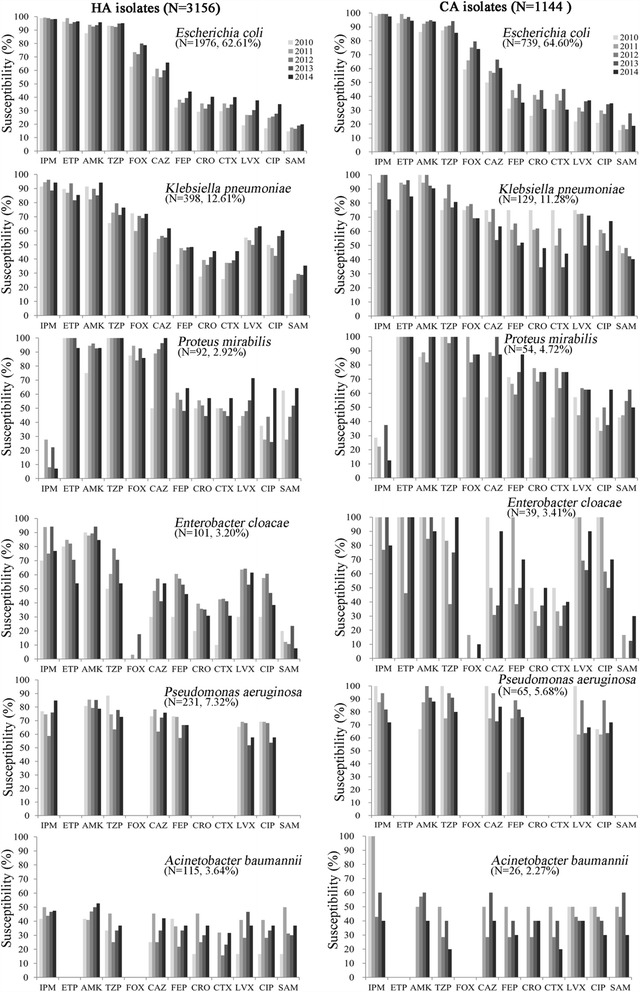
Trends over time in the susceptibility of isolates from UTIs to antimicrobial agents in China (CA and HA). *EPM, ertapenem; IPM, imipenem; AMK, amikacin; TZP, piperacillin-tazobactam; FOX, cefoxitin; FEP, cefepime; CAZ, ceftazidime; CRO, ceftriaxone; CTX, cefotaxime; LVX, levofloxacin; CIP, ciprofloxacin; SAM, ampicillin-sulbactam. Note: The data of ETP FOX CRO and CTX susceptibilities for *P. aeruginosa* and ETP as well as FOX sensitivities for *A. baumannii* were not shown because of lack of corresponding breakpoints

Figure 2d-e shows the ESBL rates in *E. coli, K. pneumonia, and P. mirabilis* from UTIs in different regions in China. We categorized the 21 participating sites into 5 different regions in China (Northeast (Haerbin, Changchun and Shenyang), North (Beijing and Tianjing), East (Hangzhou, Nanjing, Jinan, Nanchang and Shanghai), South (Chongqing, Guangzhou and Haikou) and Central China (Changsha, Zhengzhou and Wuhan)). The two sites in the Central China region exhibited higher ESBL rates in *E. coli* (81.5%) and *K. pneumonia* (64.9%), while other regions showed relatively lower ESBL rates in these two species (54.5–65.1% for *E. coli*, and 48.1–56.3% for *K. pneumoniae*). For *P. mirabilis*, the ESBL rates ranged from 31.4% (South China region) to 47.5% (North China region).

## Discussion

Nitrofurantoin, trimethoprim-sulfamethoxazole, fosfomycin, fluoroquinolones and beta-lactams are commonly recommended antimicrobial agents for urinary tract infections [14]. However, fosfomycin and nitrofurantoin are not often used in China [2]. The usage of trimethoprim-sulfamethoxazole for the treatment of UTIs in China is also limited because of a high resistance rate to this agent among *E.coli* isolates [15]. In view of this finding, we focused on the activity of beta-lactams, fluoroquinolones and aminoglycoside against uropathogens in the present study. Since *Enterobacteriaceae* accounted for the majority of aerobic and facultative anaerobic pathogens causing UTIs (88.5% of all isolates) in our study, with *E.coli*, *K. pneumonia*, *P. mirabilis* and *Enterobacter cloacae* the most frequently isolated species, knowledge of their resistance pattern is beneficial.

Cephalosporins are commonly recommended as empirical choices for UTIs, but their efficacy is greatly reduced when the pathogens produce ESBL*.* Over the entire study period, susceptibility rates of *Enterobacteriaceae* to third-generation and fourth-generation cephalosporins were 51.4–66.0% for ceftazidime, 29.4–46.9% for cefotaxime, 29.9–41.2% for ceftriaxone and 35.1–47.1% for cefepime, indicating that these agents might not be the optimum medications for empirical UTI therapies. In the present study, the percentage of ESBL positive *E. coli* isolates decreased from 66.9% in 2010 to 59.1% in 2014, while for *K. pneumonia* it decreased from 59.7 to 48.8% and from 40.0 to 26.1% among *P. mirabilis*. The data were well matched with the non-susceptibility rates to cephalosporins against each species, which indicated that ESBL production might be a reason for cephalosporin resistance [16]. The decrease of ESBL rates in *E. coli*, *K. pneumonia* and *P. mirabilis* may have been a result of China’s antimicrobial stewardship policy on antimicrobial use, which has been promoted for a number of years [17–19]. Our study also highlighted the variation in ESBL rates in different regions of China, with the Central-China region having a higher ESBL prevalence in *E. coli* and *K. pneumonia*. Researchers previously reported that the ESBL genotypes in China were mainly CTX-M types [20–22], especially CTX-M-14, −15, and −55 for *E. coli* and *K. pneumonia*, and CTX-M-65 and −14 for *P. mirabilis* [22]. Plasmids encoding these CTX-M enzymes reached human opportunists, where they have proliferated in community *E. coli* and hospital *K.* species. CTX-M families are dominate in different regions: CTX-M-15 is predominant in most of Europe, North America, the Middle East, and India, but CTX-M-14 is most common in China, Southeast Asia and Spain, while CTX-M-2 is predominant in Argentina, Israel, and Japan [23, 24]. Increased numbers of enzyme types and prevalence made determination of resistance profiles more complicated.

Fluoroquinolones, especially ciprofloxacin and levofloxacin, were considered to be effective antimicrobial agents against uropathogens because of high drug concentrations are reached in the urine. However, fluoroquinolone-resistant *E. coli* is also problematic in China. The susceptibility of *E. coli* to fluoroquinolones (ciprofloxacin and levofloxacin) was 26.9–28.9%, with rates of 30.2–32.1% against CA isolates and 25.7–27.8% against HA isolates. Wang et al. also previously reported about ciprofloxacin-resistant *E. coli* strains with multiple gyrA and parC gene substitutions [25]. Regarding the low effectiveness of fluoroquinolones against *Enterobacteriaceae*, ciprofloxacin and levofloxacin should not be considered as first line agents for empirical therapies of complicated UTIs. Our data also showed that susceptibilities of ESBL-producing *E. coli* and *K. pneumonia* strains to fluoroquinolones were significantly lower than that of ESBL-non-producing strains, which is in agreement with previous findings [26].

Carbapenems can still be considered to be suitable for severe infections and as alternative empiric treatment for UTIs caused by bacterial strains highly suspicious of being ESBL-producing or AmpC-derepressed *Enterobacteriaceae* [27–29]. Although carbapenems were not the first line choices for uncomplicated cystitis and pyelonephritis in women according to the IDSA guideline, they were good alternatives against multidrug resistant Gram-negative bacilli that caused UTIs. Our study showed that ertapenem and imipenem were the most effective agents against *Enterobacteriaceae* causing UTIs, with susceptibility rates of 92.5–96.5% and 89.9–95.2%, respectively (2010–2014). On the other hand, carbapenem-resistant *Enterobacteriaceae* have emerged, which has also been noted in other reports [30–33], especially KPC-producing *K. pneumonia* in the north-eastern area of the United States of America [31], KPC/VIM-producing *Enterobacteriaceae* in Greece [32, 33] and KPC-producing isolates in eastern China. In our study, very few *E. coli* isolates (<4%) were non-susceptible to carbapenems, while there was a certain proportion of carbapenem-non-susceptible *K. pneumonia* isolates (13.8% to ertapenem), *P. mirabilis* (85% to imipenem) and *E. cloacae* (21.3% to ertapenem and 14.9% to imipenem), which should be noted by clinicians. Especially for *E. cloacae* the susceptibility of HA samples to ertapenem has dropped to 53.9%, while for CA UTIs its susceptibility rate is 100%. Hospital infections caused by *E. cloacae*, which is a typical commensal under normal conditions, have been suggested to be mainly caused by endogenous translocation from the digestive tract in debilitated patients and that under antibiotic therapy, *E. cloacae* strains may selectively reproduce excessively in the gastrointestinal tract [34]. This might be the reason for the high ertapenem resistance in UTIs mainly caused by HA *E. cloacae*. The main resistance mechanism to carbapenem in *Enterobacteriaceae* was reported to be carbapenemase production and porin loss in China [35]. However, the resistance of *P. mirabilis* to imipenem was caused by a mechanism other than carbapenemase [13].

Among the tested antimicrobial agents, amikacin exhibited good activity against most of the uropathogens (80.0–96.2% susceptibility rate against *Enterobacteriaceae* and 83.6% against *P. aeruginosa*). Although the use of this aminoglycoside is limited because of its toxicity, it has also been recommended as an alternative to carbapenems against ESBL-producing isolates that cause UTIs [36].

## Conclusion

Carbapenems remain the most effective antimicrobial agents against UTI Gram-negative pathogens, followed by amikacin and piperacillin-tazobactam in China between 2010 and 2014. Due to the reduced susceptibility of *Enterobacteriaceae* to cephalosporins and fluoroquinolones, we recommend that these antibiotics should not be used for empirical UTI therapies in China.

## Abbreviations

AMK: Amikacin
CA: Community acquired
CAZ: Ceftazidime
CIP: Ciprofloxacin
CLSI: Clinical and Laboratory Standards Institute
CRO: Ceftriaxone
CTX: Cefotaxime
EPM: Ertapenem
ESBLs: Extended spectrum beta-lactamases
FEP: Cefepime
FOX: Cefoxitin
HA: Hospital acquired
IPM: Imipenem
LVX: Levofloxacin
MICs: Minimum inhibitory concentrations
SAM: Ampicillin-sulbactam
SMART: Study for monitoring antimicrobial resistance trends
TZP: Piperacillin-tazobactam
UTIs: Urinary tract infections
